# Tracking the Emergence of Azithromycin Resistance in Multiple Genotypes of Typhoidal *Salmonella*

**DOI:** 10.1128/mBio.03481-20

**Published:** 2021-02-16

**Authors:** Mohammad S. I. Sajib, Arif M. Tanmoy, Yogesh Hooda, Hafizur Rahman, Jason R. Andrews, Denise O. Garrett, Hubert P. Endtz, Samir K. Saha, Senjuti Saha

**Affiliations:** a Child Health Research Foundation, Dhaka, Bangladesh; b Institute of Biodiversity, Animal Health, and Comparative Medicine, University of Glasgow, Glasgow, United Kingdom; c Department of Medical Microbiology and Infectious Diseases, Erasmus University Medical Center, Rotterdam, The Netherlands; d MRC-Laboratory Molecular Biology, Cambridge, United Kingdom; e Division of Infectious Diseases and Geographic Medicine, Stanford University School of Medicine, Stanford, California, USA; f Sabin Vaccine Institute, Washington, DC, USA; g Fondation Mérieux and Centre International de Recherche en Infectiologie, INSERM, Lyon, France; h Department of Microbiology, Dhaka Shishu Hospital, Dhaka, Bangladesh; i Bangladesh Institute of Child Health, Dhaka, Bangladesh; Yale School of Public Health

**Keywords:** typhoid, *Salmonella* Typhi infection, paratyphoid fever, antimicrobial drug resistance, azithromycin, Bangladesh, AMR, Paratyphi, Typhi, paratyphoid, typhoid

## Abstract

The rising prevalence of antimicrobial resistance in Salmonella enterica serovars Typhi and Paratyphi A, causative agents of typhoid and paratyphoid, have led to fears of untreatable infections. Of specific concern is the emerging resistance against azithromycin, the only remaining oral drug to treat extensively drug resistant (XDR) typhoid. Since the first report of azithromycin resistance from Bangladesh in 2019, cases have been reported from Nepal, India, and Pakistan. The genetic basis of this resistance is a single point mutation in the efflux pump AcrB (R717Q/L). Here, we report 38 additional cases of azithromycin-resistant (AzmR) *Salmonella* Typhi and Paratyphi A isolated in Bangladesh between 2016 and 2018. Using genomic analysis of 56 AzmR isolates from South Asia with AcrB-R717Q/L, we confirm that this mutation has spontaneously emerged in different *Salmonella* Typhi and Paratyphi A genotypes. The largest cluster of AzmR Typhi belonged to genotype 4.3.1.1; Bayesian analysis predicts the mutation to have emerged sometime in 2010. A travel-related Typhi isolate with AcrB-R717Q belonging to 4.3.1.1 was isolated in the United Kingdom, increasing fears of global spread. For real-time detection of AcrB-R717Q/L, we developed an extraction-free, rapid, and low-cost mismatch amplification mutation assay (MAMA). Validation of MAMA using 113 AzmR and non-AzmR isolates yielded >98% specificity and sensitivity versus phenotypic and whole-genome sequencing assays currently used for azithromycin resistance detection. With increasing azithromycin use, AcrB-R717Q/L is likely to be acquired by XDR strains. The proposed tool for active detection and surveillance of this mutation may detect pan-oral drug resistance early, giving us a window to intervene.

## INTRODUCTION

Salmonella enterica serovars Typhi and Paratyphi A, which cause typhoid and paratyphoid, respectively, are estimated to be responsible for 14.3 million illnesses and 136,000 deaths globally each year (1). The majority of the burden lay disproportionately on low- and middle-income countries (LMICs), specifically South Asia and Sub-Saharan Africa. Typhoidal *Salmonella* has exhibited resistance to all antimicrobials widely used to treat typhoid and paratyphoid, leaving limited options for treatment and raising fears of untreatable infections.

In the early 1900s, mortality due to typhoid/paratyphoid exceeded 30% in many areas, but ampicillin, chloramphenicol, and trimethoprim-sulfamethoxazole were instrumental in reducing it to <1% (2–4). Simultaneous resistance to all three first line of drugs, defined as multidrug resistance (MDR), started emerging in the 1980s; MDR strains carried an IncHI1 plasmid with multiple resistance genes (5–7). As a result, the primary treatment for enteric fever shifted to fluoroquinolones, but soon there were reports of decreasing fluoroquinolone susceptibility due to the rise of point mutations in the gyrase and topoisomerase genes (8, 9). Finally, in addition to a few sporadic reports of ceftriaxone resistance (10, 11), in 2016, an outbreak of extensively drug-resistant (XDR) *Salmonella* Typhi (resistant to chloramphenicol, ampicillin, trimethoprim-sulfamethoxazole, streptomycin, fluoroquinolones, and third-generation cephalosporins) was identified in Pakistan; to date, more than 11,000 cases have been confirmed (12). Cephalosporin resistance of the XDR strains is caused by the acquisition of a broad-spectrum beta-lactamase resistance gene on a plasmid (13). For patients with uncomplicated XDR typhoid in Pakistan, azithromycin is the last oral option. With the increasing use of this antimicrobial in South Asia, the number of reports of azithromycin-resistant (AzmR) *Salmonella* Typhi is on the rise.

Our group demonstrated that the underlying mechanism of resistance is a point mutation at amino acid position 717 (R717Q/L) in the efflux pump encoded by the *acrB* gene (14). The presence of this single nucleotide polymorphism (SNP) raises the MICs of *Salmonella* Typhi and Paratyphi A for azithromycin to ≥32 μg/ml, the Clinical and Laboratory Standards Institute (CLSI) breakpoint of resistance (15). Since the report from Bangladesh in 2019, at least six AzmR *Salmonella* Typhi cases mediated by this mutation have been reported from Nepal, India, and Pakistan, the epicenter of the XDR typhoid outbreak (16–18). However, no further AzmR *Salmonella* Paratyphi A has been described. This makes it imperative to gain deeper insights into the evolution and spread of this mutation in typhoidal *Salmonella*. Low-cost and rapid diagnostic methods to identify this mutation need to be developed, since it can currently only be identified using whole-genome sequencing (WGS).

Leveraging our ongoing enteric fever surveillance system (19), which yielded 3,025 *Salmonella* Typhi and Paratyphi A isolates since 2016 in Bangladesh, and the available public data of other reports of AzmR strains from the region, we used WGS and comparative genomics to understand the emergence, evolution, and spread of azithromycin resistance conferred for the AcrB-R717 mutation. In addition, we developed a PCR-based mismatch amplification mutation assay (MAMA) to detect AcrB-R717 mutations in typhoidal *Salmonella* serovars, which is a low-cost and straightforward tool to rapidly identify and track this mutation.

## RESULTS

### Azithromycin resistance in Bangladesh.

Between October 2016 and July 2018, we screened 2,519 isolates of *Salmonella* Typhi and 506 *Salmonella* Paratyphi A for azithromycin resistance using disc diffusion and identified 59 *Salmonella* Typhi and 45 *Salmonella* Paratyphi A potentially resistant isolates (zone diameters of ≤12 mm [15]). Further assessment of these 104 isolates by Etest identified 32 *Salmonella* Typhi and 6 *Salmonella* Paratyphi A isolates with MIC ≥32 μg/ml, confirming them as AzmR isolates (20). In addition, we identified two *Salmonella* Typhi and three *Salmonella* Paratyphi A isolates with MICs of 24 μg/ml; these isolates are not considered AzmR by the CLSI guidelines, but the MIC is much higher than for other nonresistant isolates (see Table S1 in the supplemental material).

10.1128/mBio.03481-20.4 TABLE S1 Details of 113 *Salmonella* Typhi and Paratyphi A isolates detected in Bangladesh and used to validate the mismatch amplification mutation assay. Download Table S1, XLSX file, 0.01 MB. Copyright © 2021 Sajib et al. 2021 Sajib et al. https://creativecommons.org/licenses/by/4.0/ This content is distributed under the terms of the Creative Commons Attribution 4.0 International license.

We conducted WGS analyses for all 32 AzmR *Salmonella* Typhi and 6 AzmR Paratyphi A isolates, as well as the 5 non-AzmR isolates with high MICs. All 32 AzmR *Salmonella* Typhi isolates had the AcrB-R717Q/L mutation; 29 had AcrB-R717Q, and three had AcrB-R717L. Five Paratyphi A isolates contained AcrB-R717Q. Of the five non-AzmR isolates with a high MIC (24 μg/ml), one *Salmonella* Typhi also contained an AcrB-R717Q mutation, whereas the other four did not. For downstream analysis of Bangladeshi isolates, we included the 38 isolates with the AcrB-R717Q/L mutation identified here (including the one with an MIC of 24 μg/ml) and another 13 isolates from our previous study on the emergence of azithromycin resistance in Bangladesh during 2009 to 2016, taking the total AzmR-resistant isolates identified in Bangladesh to 51.

**FIG 1:**
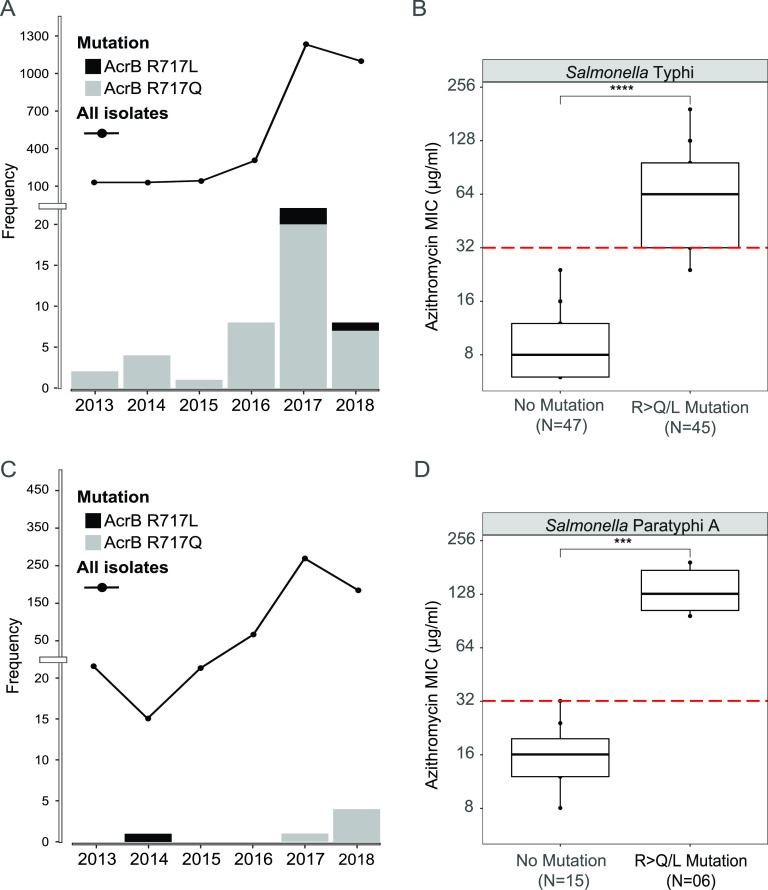
Detection of azithromycin resistance and types of AcrB-717 mutation in *Salmonella* Typhi and Paratyphi A in Bangladesh. (A) Temporal distribution of 3043 *Salmonella* Typhi isolates identified in this study and by Hooda et al. (14). The total number of isolates tested is shown as the line plot from 2013 to 2018. The numbers of AzmR strains isolated each year is shown in the bar plot. (B) Azithromycin MICs among AcrB-WT and AcrB-R717Q and AcrB-R717QL mutant strains of *Salmonella* Typhi. (C) Temporal distribution of 587 *Salmonella* Paratyphi A isolates identified in this study and by Hooda et al. (14). The number of isolates is shown as the line plot from 2013 to 2018. The number of AzmR strains isolated each year is shown in the bar plot. (D) Azithromycin MICs among *acrB* wild-type and AcrB-R717Q and AcrB-R717QL mutant strains of *Salmonella* Paratyphi A. ****, *P* ≤ 0.0001; ***, *P* ≤ 0.001.

**FIG 2:**
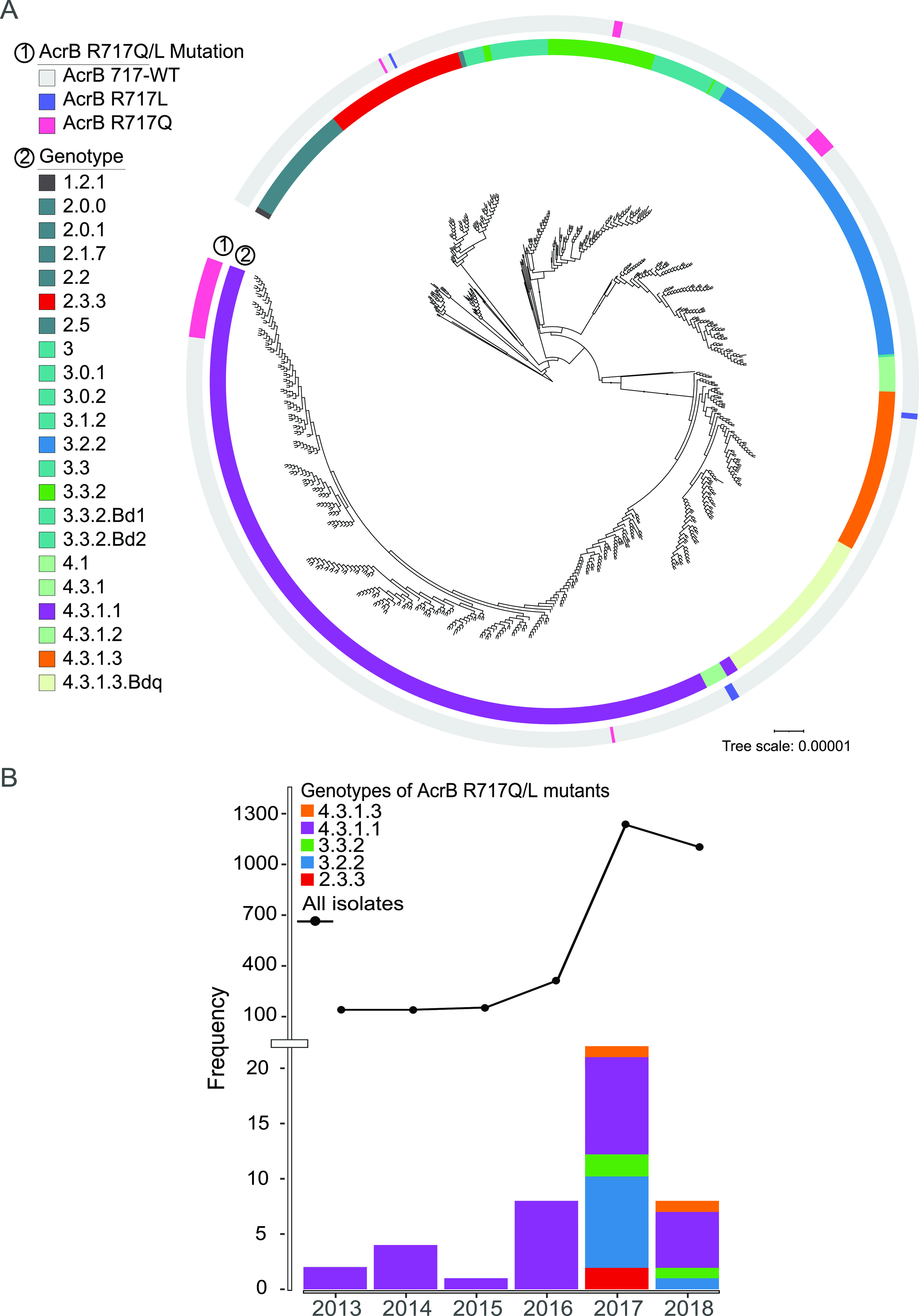
Spontaneous emergence of AcrB-R717Q/L mutation in five different genotypes of *Salmonella* Typhi. (A) A whole-genome SNP tree containing 825 *Salmonella* Typhi strains highlights different azithromycin-resistant *Salmonella* Typhi genotypes. The inner circle shows the distribution of *Salmonella* Typhi genotypes, and the outer circle shows the mutations in AcrB-717. (B) Temporal distribution of the *acrB* mutation in different genotypes between 2013 and 2018.

The first *Salmonella* Typhi with an AcrB-R717Q/L mutation was isolated in 2013. Since then, there has been a gradual increase in the number of AzmR *Salmonella* Typhi isolates. The number of total Typhi/Paratyphi A isolates also increased over the years with implementation of enhanced surveillance programs (Fig. 1A). All of the AzmR *Salmonella* Typhi strains isolated between 2013 and 2016 belonged to genotype 4.3.1.1 (Fig. 2B). Additional genotypes with the same mutations began to emerge in 2017; these included genotypes 2.3.3, 3.2.2, 3.3.2, and 4.3.1.3 (Fig. 2A and B). The difference in MICs between *Salmonella* Typhi with AcrB-R717Q/L mutations or without (AcrB-WT) was 8-fold (mean MIC = 73.77 μg/ml versus 9.48 μg/ml: Fig. 1B; see also Fig. S1A in the supplemental material). The first *Salmonella* Paratyphi A with AcrB-R717L mutation was identified in 2014. Five additional *Salmonella* Paratyphi A strains were identified in 2017 and 2018 (Fig. 1C). The mean MIC for Paratyphi A isolates with AcrB-R717L/Q is also 8-fold higher than for AcrB-WT (138.66 μg/ml versus 17.06 μg/ml [Fig. 1D; see also Fig. S1B]).

10.1128/mBio.03481-20.1 FIG S1 MICs of the 92 *Salmonella* Typhi (A) and 21 *Salmonella* Paratyphi A (B) isolates tested for azithromycin resistance using E-strips. Further details of all tested isolates are provided in Table S1. Download FIG S1, PDF file, 0.4 MB. Copyright © 2021 Sajib et al. 2021 Sajib et al. https://creativecommons.org/licenses/by/4.0/ This content is distributed under the terms of the Creative Commons Attribution 4.0 International license.

### Spontaneous appearance of AcrB-R717Q/L mutations in different *Salmonella* Typhi genotypes.

To gain further genomic and evolutionary insight into the AcrB-717 mutations of *Salmonella* Typhi, we contextualized 33 AcrB-R717Q/L and 47 AcrB-717WT isolates collected in the same study in a maximum-likelihood phylogenetic tree against 750 *Salmonella* Typhi genomes reported from Bangladesh in previous studies (14, 18, 21, 22) (see Table S2). These previous studies included 12 genomes with AcrB-R717Q/L mutations. In addition, the tree also included five AcrB-717 mutants previously identified in the United Kingdom, Nepal, and Pakistan (16–18). The final tree contained 50 *Salmonella* Typhi genomes with AcrB-R717Q/L. The largest cluster of *Salmonella* Typhi genomes with the AcrB-R717Q mutation belonged to genotype 4.3.1.1 (62%; *n* = 31), followed by 3.2.2 (18%; *n* = 9), 3.3.2 (6%; *n* = 3), and 2.3.3 (2%; *n* = 1) (Fig. 2A). Six isolates had the AcrB-R717L mutation; three belonged to genotype 4.3.1, two belonged to 4.3.1.3, and one belonged to 2.3.3 (Fig. 2A). The presence of this mutation in different genotypes indicates that AcrB-R717Q/L has emerged spontaneously and independently multiple times.

10.1128/mBio.03481-20.5 TABLE S2 Details of all 835 azithromycin-resistant and azithromycin-sensitive *Salmonella* Typhi strains used to build a maximum-likelihood phylogenetic tree. Download Table S2, XLSX file, 0.05 MB. Copyright © 2021 Sajib et al. 2021 Sajib et al. https://creativecommons.org/licenses/by/4.0/ This content is distributed under the terms of the Creative Commons Attribution 4.0 International license.

**FIG 3:**
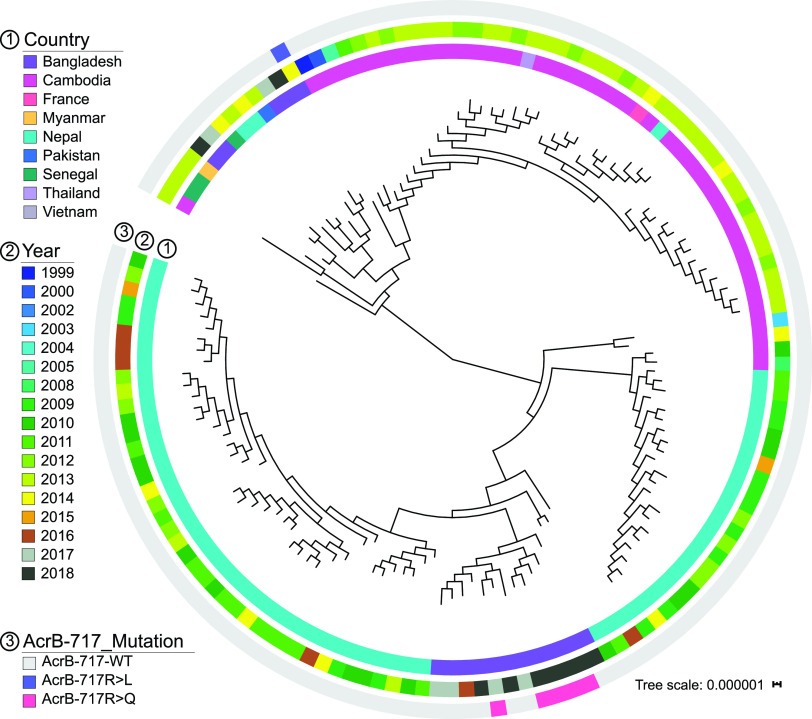
Maximum-likelihood tree based on SNP alignment of 141 *Salmonella* Paratyphi A strains with six azithromycin-resistant isolates. The inner circle depicts the country of isolation, the circle in the middle depicts the year of isolation, and the outer circle depicts the different mutations at AcrB-717.

At least two instances of the independent emergence of AcrB-R717 mutations were also observed in the phylogenetic tree of *Salmonella* Paratyphi A (Fig. 3). This tree included five AzmR and 15 randomly selected sensitive *Salmonella* Paratyphi A isolates, contextualized against 121 publicly available genomes (see Table S3) (14, 23, 24). The five *Salmonella* Paratyphi A with AcrB-R717Q isolated between 2016 and 2018 clustered together, whereas the only genome with AcrB-R717L belonged to a distant and much smaller Bangladesh-specific cluster (Fig. 3).

10.1128/mBio.03481-20.6 TABLE S3 Details of all 141 azithromycin-resistant and azithromycin-sensitive *Salmonella* Paratyphi A strains used to build a maximum-likelihood phylogenetic tree. Download Table S3, XLSX file, 0.01 MB. Copyright © 2021 Sajib et al. 2021 Sajib et al. https://creativecommons.org/licenses/by/4.0/ This content is distributed under the terms of the Creative Commons Attribution 4.0 International license.

### Emergence and transmission of AcrB-R717Q mutation within the predominant genotype 4.3.1.1.

**FIG 4:**
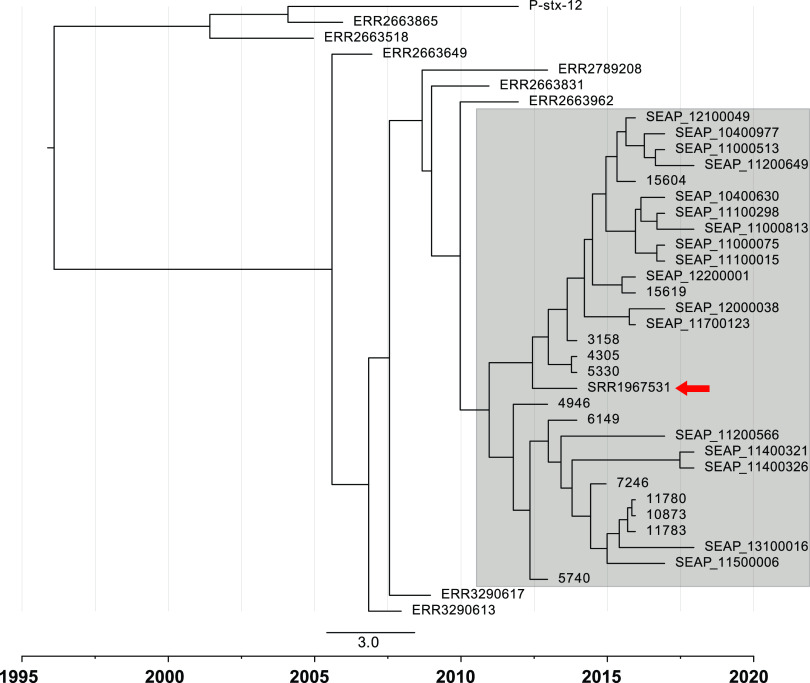
Bayesian estimation of the maximum clade credibility tree of genotype 4.3.1.1 azithromycin-resistant and related azithromycin-sensitive *Salmonella* Typhi isolates. The azithromycin-resistant clade containing the AcrB-R717Q mutation is shaded in gray, and the closely related AcrB-WT strains are outside the gray box. The *Salmonella* Typhi strain P-stx-12 (AcrB-WT) belonging to 4.3.1.1 was used as a reference strain. The AcrB-R717Q mutation is predicted to have emerged between 2010 and 2012 in Bangladesh. The travel-related *Salmonella* Typhi isolate from the United Kingdom is highlighted with a red arrow. The scale bar indicates the number of substitutions per variable site per year.

As mentioned above, the largest cluster of AzmR strains in Bangladesh belonged to genotype 4.3.1.1 (*n* = 29). In addition, the genome of *Salmonella* Typhi isolated in the United Kingdom that contained the AcrB-R717Q mutation also belonged to the same cluster, and was related to travel to Bangladesh (14, 25). To gain insights into the history of the emergence of this mutation, we performed evolutionary analysis using the Bayesian Evolutionary Analysis Sampling Trees (BEAST) program, focusing on these 30 Bangladeshi *Salmonella* Typhi genomes containing the AcrB-R717Q mutation and eight closely related genomes with AcrB-WT as identified from the phylogenetic analysis. *Salmonella* Typhi strain P-stx-12 that belongs to genotype 4.3.1.1 and has a complete annotated genome was also included. The maximum clade credibility tree obtained indicated that 4.3.1.1 AcrB-R717Q cluster emerged between 2010 and 2012 (Fig. 4), not long before it was first detected in our passive typhoid surveillance in 2013. All AzmR isolates of this cluster have four unique SNPs in genes STY2741, STY0519, and STY1399, which were also described previously (14).

### Rapid and low-cost PCR detection of AcrB-R717 mutation.

**FIG 5:**
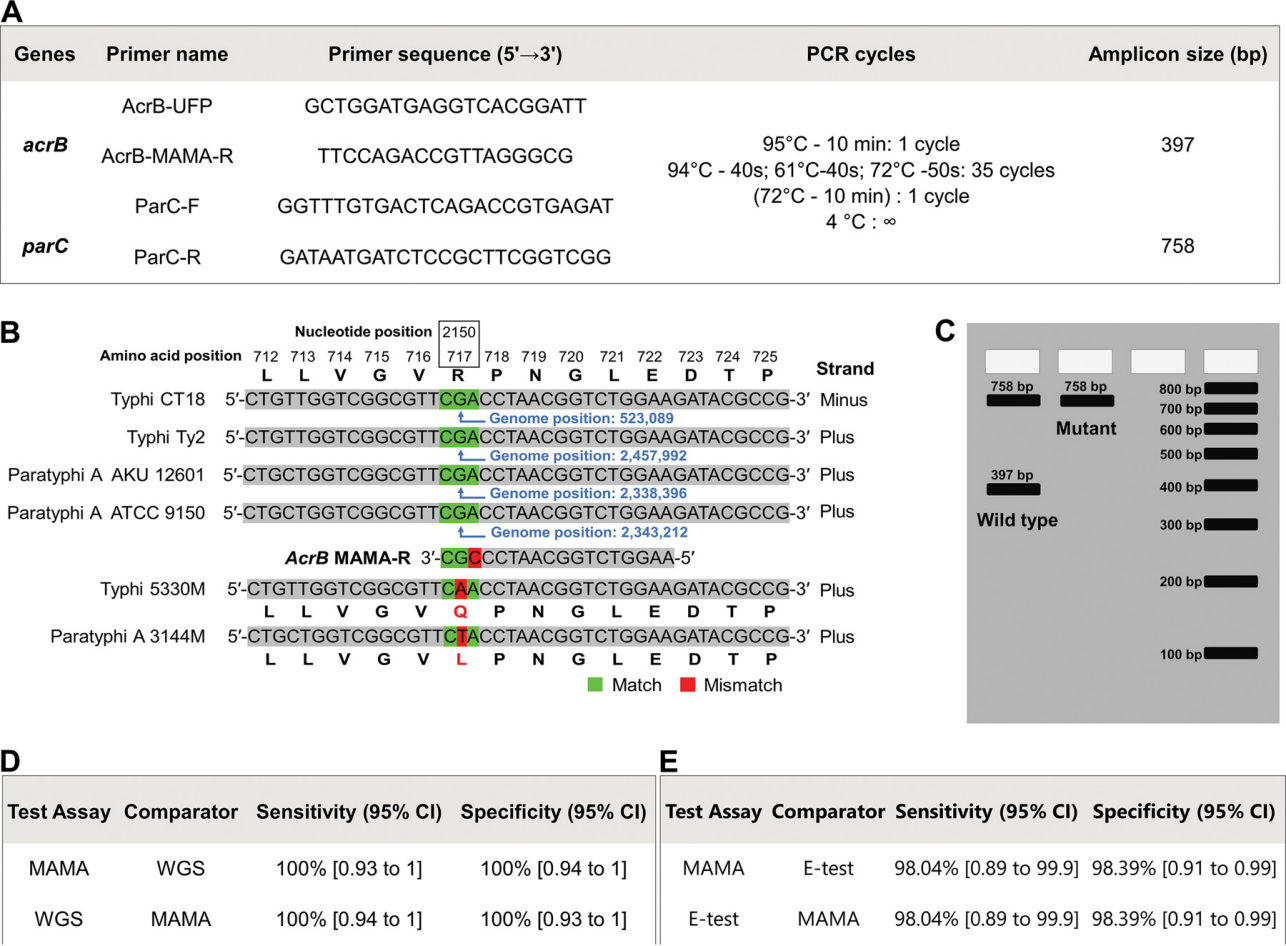
Design and sensitivity and specificity of PCR-based mismatch amplification mutation assay (MAMA) for detecting AcrB mutations. (A) PCR cycling condition, primer sequences, and amplicon sizes generated by the AcrB MAMA and ParC control primers utilized in this study. (B) MAMA PCR primers for detecting mutation at the nucleotide position 2150 (amino acid 717) of the *acrB* gene. A single mismatch was incorporated at the conserved nucleotide (A to C; red, AcrB-MAMA-R) to increase allelic discrimination and chain termination in the presence of any mutation (G to A or T; red highlight). Published sequences of typhoidal *Salmonella* (e.g., *Salmonella* Typhi 5330M and *Salmonella* Paratyphi A 3144M [14]) with both Q (glutamine) and L (leucine) mutations were used to design the assay *in silico*. (C) Interpretation of the bands generated by AcrB MAMA and ParC control primers designed in this study. (D) Sensitivity and specificity of MAMA and WGS. (E) Sensitivity and specificity of MAMA and Etest compared to each other.

For rapid and low-cost detection of azithromycin resistance in typhoidal *Salmonella*, we designed a mismatch amplification mutation assay (MAMA) to detect the SNP of the *acrB* gene that leads to the AcrB-R717Q/L mutation. The primal focus of this rapid mismatch assay was nucleotide 2150 (part of the codon for amino acid 717) of the *acrB* gene. A universal forward primer AcrB-UFP, targeting the conserved upstream region (nucleotides 1770 to 1789) of the mutation, was designed. The reverse primer AcrB-MAMA-R was designed so that it partially matches the wild-type allele of the *acrB* gene with a mismatch at the third nucleotide (A to C) from the 3′ end; this increased allelic discrimination (Fig. 5A and B). A single nucleotide discrepancy in the AcrB-MAMA-R primer has minimal impact on the PCR yield but terminates the reaction if an additional neighboring mismatch is present, such as the SNP at position 2150 (Fig. 5B and C) (26, 27). A control set of primers (ParC-F and ParC-R) targeting the highly conserved *parC* (topoisomerase IV) gene of *Salmonella* Typhi and *Salmonella* Paratyphi A was also designed as a PCR control so that it can be run in a multiplex fashion with the AcrB primers described above (Fig. 5A). Further details of this PCR are provided in Materials and Methods. The entire process of detection of the mutation using this procedure with an isolate takes ∼3.5 h; the step of boiling and centrifugation takes ∼25 min, reaction mix preparation and PCR takes ∼120 min, and gel preparation and electrophoresis takes ∼60 min.

To validate the assay, 51 *Salmonella* Typhi and Paratyphi A AcrB-R717Q/L mutants (38 found in this study and 13 reported by Hooda et al. [14]) and 62 randomly selected isolates (47 *Salmonella* Typhi and 15 *Salmonella* Paratyphi A) with AcrB-WT were chosen from our collection (see Table S1). PCR using AcrB-UFP and AcrB-MAMA-R primers generated a 397-bp amplicon in wild-type (WT) strains. As expected, there was no band for AcrB mutants due to PCR inhibition. In all cases, ParC control primers yielded a 758-bp band indicating successful PCR amplification (see Fig. S2). The test set of 113 *Salmonella* Typhi and Paratyphi A AcrB R717Q/L mutants and WT isolates detected via MAMA exhibited 100% sensitivity and 100% specificity compared to WGS data (Fig. 5D) and 98% sensitivity and 98.4% specificity compared to Etest results (Fig. 5E). All PCRs for validation were conducted using boiled DNA, bypassing the step of DNA extraction to reduce time and cost and to save resources.

10.1128/mBio.03481-20.2 FIG S2 Gel electrophoresis image of the bands generated by AcrB MAMA and ParC control primers designed in this study. Wild-type strains produce a 397-bp band alongside the ParC internal control band of 758 bp. AcrB-R717Q/L mutants produce no band except the 758-bp ParC internal control. Download FIG S2, PDF file, 0.6 MB. Copyright © 2021 Sajib et al. 2021 Sajib et al. https://creativecommons.org/licenses/by/4.0/ This content is distributed under the terms of the Creative Commons Attribution 4.0 International license.

## DISCUSSION

The rising prevalence of resistance and the drying pipeline of new antimicrobials have left us with a few options of oral drugs to treat typhoidal *Salmonella*. In this study, we describe the increasing resistance of *Salmonella* Typhi and Paratyphi A against azithromycin, the last oral antimicrobial against uncomplicated typhoid fever, in Bangladesh and other countries of endemicity. Since the first confirmed identification of AzmR *Salmonella* Typhi, conferred by a SNP, from Bangladesh, there has been an increasing number of reports from surrounding countries in South Asia. With the largest collection of AzmR isolates, our data, in concordance with other publications, show that azithromycin resistance has arisen independently multiple times in different genotypes in *Salmonella* Typhi. We further go on to show similar spontaneous emergence in Paratyphi A isolates. In order to vigilantly track this mutation and guide treatment options and decisions, we developed a low-cost PCR tool for quick detection of the mutation, AcrB-R717Q/L, responsible for azithromycin resistance.

Since 2013, we have identified over 50 Typhi and *Salmonella* Paratyphi A isolates in Bangladesh that carried the AcrB-R717Q/L mutation and were resistant to azithromycin. Phylogenetic analysis showed the presence of this SNP in several different genotypes, indicating independent emergence of the AcrB-R717 mutation in both *Salmonella* Typhi and Paratyphi A (Fig. 2A and 3). In *Salmonella* Typhi, we also noted that the diversity of genotypes carrying the AzmR mutation increases over time, most likely due to the strong selection pressure placed by increasing use of azithromycin. Use of this antimicrobial is common in many countries of endemicity, including Pakistan and Bangladesh, where the drug is sold over-the-counter without a prescription (28).

In Bangladesh, the AcrB-R717Q/L mutation is most frequently observed in *Salmonella* Typhi genotype 4.3.1.1 (see Fig. S3), which is also the predominant circulating genotype in the country (Fig. 2A) (22). Using Bayesian analysis, we predict that the first AcrB-R717 mutation in this genotype appeared between 2010 and 2012, which subsequently led to the detection of 29 cases in our surveillance in Dhaka, Bangladesh (14). A travel-related AzmR isolate identified in the United Kingdom belonged to the same cluster, confirming the fear of global transmission of this mutation. In addition, the XDR *Salmonella* Typhi outbreak strain of Pakistan also belongs to genotype 4.3.1.1 (13), and recently, the same AcrB mutation was identified in this genotype in Pakistan (17). Fortunately, the Pakistani AzmR strain was not XDR, but the gradual takeover of the entire *Salmonella* Typhi population by this genotype heralds the emergence of pan-oral drug-resistant (PoDR; resistant to chloramphenicol, ampicillin, trimethoprim-sulfamethoxazole, fluoroquinolones, third-generation cephalosporins, and azithromycin) *Salmonella* Typhi unless new effective oral drugs are widely introduced (29).

10.1128/mBio.03481-20.3 FIG S3 Spatial distribution of azithromycin-resistant strains in the Dhaka district, Bangladesh. (A) *Salmonella* Typhi genotypes with AcrB-R717Q/L mutation in Dhaka (*n* = 42) colored by genotype. (B) *Salmonella* Typhi with AcrB-R717Q/L mutation in Dhaka from 2013 to 2018 (*n* = 42) colored by year of isolation. (C) *Salmonella* Paratyphi A with AcrB-R717Q/L mutation in Dhaka from 2013 to 2018 (*n* = 6) colored by year of isolation. Download FIG S3, PDF file, 1.5 MB. Copyright © 2021 Sajib et al. 2021 Sajib et al. https://creativecommons.org/licenses/by/4.0/ This content is distributed under the terms of the Creative Commons Attribution 4.0 International license.

Monitoring this AcrB mutation is essential to track its spread and forecast PoDR typhoid outbreaks. Currently, the only method of detecting this mutation is by WGS, which is cost and resource extensive and not possible to continuously conduct in LMICs in real-time to guide rapid public health action. To overcome this barrier, we developed a mismatch amplification mutation assay that can reliably detect the AcrB-717 mutation using a simple step of conventional PCR, without the requirement of the time-consuming and expensive steps of DNA extraction. It costs <USD 1.5, requires a conventional PCR machine, demands little expertise, and can be performed in a basic laboratory, delivering results in ∼3.5 h. We validated this method by using a mix of 113 resistant and sensitive *Salmonella* Typhi and Paratyphi A isolates, and this assay showed 100% concordance with the WGS data. Compared to Etest results, it showed more than 98% sensitivity and specificity.

Considering the quick turnaround times, high sensitivity, and specificity of this test, it may be used in accompaniment with phenotypic susceptivity tests for azithromycin, which takes >16 h after isolation of the bacteria (∼18 h). In addition, some laboratories are unable to conduct MIC tests as E-strips are expensive and, as shown in this and previous studies, the results of disk diffusion assays of azithromycin are unreliable (only 36.5% are true resistant isolates) (14). The interpretation of the PCR assay is simple, requiring no additional training: the presence or absence of a 397-nucleotide band indicates the absence or presence of the mutation. We have also incorporated an internal control using the conserved gene *parC* to confirm valid PCR runs. It must be noted, however, that (i) this assay cannot distinguish between the two mutations (R717Q and R717L), which are also phenotypically indistinguishable, and (ii) new modes of azithromycin resistance will not be detected through this method.

This study highlights the increasing and spontaneous occurrence of azithromycin resistance in both *Salmonella* serovars Typhi and Paratyphi A. Although no azithromycin-resistant XDR isolate has been reported to date, (i) the increasing use of azithromycin, (ii) the spontaneous acquisition of AcrB-R717 mutations, (iii) the clear historical record of widespread dissemination of resistance to all previously widely used antimicrobials by *Salmonella* Typhi, and (iv) the global spread of XDR strains (3, 4, 44) suggest that strains resistant to almost all oral antimicrobials is only a matter of time. Acquisition of the plasmid that confers cephalosporin resistance in XDR strains by the Bangladeshi AzmR strains or acquisition of the AcrB mutation in the XDR strains in Pakistan could be the end of oral treatment for typhoid. After fluoroquinolone-nonsusceptible isolates appeared, they became the dominant strains within a decade. Similarly, XDR *Salmonella* Typhi isolates now account for >70% of *Salmonella* Typhi isolated in Pakistan, less than 5 years after their first appearance (30, 31). This underscores the risk of PoDR *Salmonella* Typhi becoming the dominant type in the near future once it appears. This would pose serious threats to the health system of LMICs, where typhoid is endemic. Institution of immediate preventative measures, such as improved water, sanitation, and hygiene, and active AMR surveillance to track the resistant genotypes are imperative to interrupt this trend toward the emergence and spread of PoDR.

## MATERIALS AND METHODS

### Study site and patient enrollment.

In this study, we report data from enteric fever surveillance conducted in the inpatient departments of the two largest pediatric hospitals of Bangladesh—Dhaka Shishu (Children) Hospital and Shishu Shasthya (Child Health) Foundation Hospital—and three branches of an outpatient-based clinic, the Popular Diagnostic Center. These sites include sentinel sites of the World Health Organization-supported Invasive Bacterial Vaccine Preventable Diseases surveillance platform (32) and the Surveillance of Enteric Fever Project of Sabin Vaccine Institute (33).

### Etiology detection and antimicrobial susceptibility testing.

Identification of blood culture positive *Salmonella* Typhi/Paratyphi A isolates was first confirmed by standard biochemical tests. Next, all *Salmonella* species were confirmed using polyvalent *Salmonella* O antisera (A–S MAST Assure; Mast Group, Ltd., Liverpool, UK). All Typhi and Paratyphi A serovars were confirmed using the monovalent antisera factor-d and factor-O2 (MAST Assure), respectively, accompanied by *Salmonella*-specific antisera. For antimicrobial susceptibility testing (AST), the Kirby-Bauer disk diffusion method was followed utilizing 15-μg azithromycin discs (Oxoid, Thermo Scientific, Waltham, MA) (34). To determine azithromycin MIC, E-strips were used (bioMérieux, France). Since there is no defined azithromycin CLSI breakpoint for any *Salmonella* serovars other than *Salmonella* Typhi, the following definition of resistance (adopted from *Salmonella* Typhi) was used: zone ≤ 12 mm and MIC ≥ 32 μg/ml (20). ASTs were performed in a blinded fashion without any prior knowledge of sequence data to minimize bias.

### DNA extraction and whole-genome sequencing.

Isolates were grown on MacConkey agar (Oxoid, UK) overnight and DNA was extracted from a suspension of the overnight culture using the QIAamp DNA minikit (Qiagen, Hilden, Germany). Sequencing was conducted in an Illumina HiSeq 4000 paired-end (150-bp) system at the Wellcome Sanger Institute. Raw reads of all the isolates sequenced in this study can be found under ENA study accession number PRJEB30334.

### Acquiring additional genomic data, SNP-based phylogenetic analysis, and detection of AcrB-717 mutations.

A total of 835 *Salmonella* Typhi isolates were used for phylogenetic analysis; 755 genomes were described elsewhere, and 80 were sequenced here (see Table S2) (14, 15, 21, 22, 35). Phylogenetic analyses of *Salmonella* Paratyphi A included 141 isolates (14 were sequenced in this study, and 121 were described elsewhere) (see Table S3) (14, 36, 37).

Raw fastq reads were mapped using Bowtie2 (default options) against the CT18 reference genome (GenBank accession AL513382.1) for *Salmonella* Typhi or, AKU_12601 (GenBank accession FM200053.1) for *Salmonella* Paratyphi A. Candidate SNPs were identified using SAMtools (*view -ubS; sort; index; mpileup -d 1000 -t DP -t SP -ugBf*) and bcftools (*call -cv; view -v snps*) (38). Only the homozygous (*bcftools view -g hom*), unambiguous SNPs with a phred-quality score of >20 were selected using a customized python script (https://github.com/CHRF-Genomics/filter_SNP_quality). SNPs were discarded if they had strand bias of *P* < 0.001, a mapping bias of *P* < 0.001, or a tail bias of *P* < 0.001 (*vcfutils.pl varFilter -1 0.001-3 0.001-4 0.001*). SNPs located in phage or repeat regions (354 kb for *Salmonella* Typhi CT18 as reported earlier [39] and 118.9 kb for *Salmonella* Paratyphi AKU_12601 as detected in PHASTER and GenBank annotation filtered with a customized python script [https://github.com/katholt/genotyphigithub.com/CHRF-Genomics/extract_position_from_GenBank]) were also excluded. Gubbins was used (*run_gubbins –tree_builder raxml –converge_method recombination –raxml_model GTRGAMMA –verbose*) to detect the recombinant regions (40), and SNPs in these regions were excluded as well, resulting in final alignments of 4,888 and 1,042 chromosomal SNPs for *Salmonella* Typhi and Paratyphi. All *Salmonella* Typhi strains were genotyped using the genotyphi script (https://github.com/katholt/genotyphi). The Bdq sublineage of genotype 4.3.1.3 was detected using another script (https://github.com/arif-tanmoy/DetectBdq). A customized python script was written to detect the mutation at AcrB-717 in *Salmonella* Paratyphi A (github.com/CHRF-Genomics/paratyphiA_acrB717_screening).

Maximum-likelihood trees (MLT) were built from the chromosomal SNP alignments using RAxML version 8.2.12 (with the Generalized Time-Reversible model and a Gamma distribution to model site-specific rate variation [GTRGAMMA] in RAxML) (41). Support for the MLT was calculated using 100 bootstrap pseudoanalyses of the alignment. The MLT was outgroup rooted by including the pseudoalleles from *Salmonella* Paratyphi A AKU_12601 (for *Salmonella* Typhi) or *Salmonella* Typhi CT18 (for *Salmonella* Paratyphi A) in the alignment. Tree visualization was performed using iTol v5.5 (42). The same tool was used to prune 10 *Salmonella* Typhi with low genotyping support (<0.9) from the MLT of *Salmonella* Typhi, eventually making it an MLT of 825 isolates.

### Evolutionary analysis with BEAST.

Bayesian phylogenetic analyses were conducted to investigate the emergence of AcrB-R717Q mutation in isolates belonging to genotype 4.3.1.1. The 30 AzmR isolates from Bangladesh and 8 closely related azithromycin-sensitive *Salmonella* Typhi genomes belonging to 4.3.1.1 were selected (see Table S4), and SNPs were identified using the ParSNP tool (+phipack package) (43) with *Salmonella* Typhi P-stx-12 strain as the reference genome (44). The SNPs were subsequently used for Bayesian analysis with BEAST v1.10.4 (45). GTR+G_4_ substitution model, an uncorrelated lognormal relaxed-clock model, and the exponential coalescent tree prior were used. Three independent analyses were performed with 5 × 10^8^ steps, recording samples every 5 × 10^4^ steps. The results from the three independent analyses gave similar estimates for the emergence of the 4.3.1.1 AzmR cluster. To calibrate the molecular clock, we used the sampling year of all sequences. The selected model combinations, including the molecular clock model and tree priors, have previously been used by Park et al. (39) for *Salmonella* Typhi evolutionary analysis.

10.1128/mBio.03481-20.7 TABLE S4 Details of 39 azithromycin-resistant and azithromycin-sensitive *Salmonella* Typhi strains belonging to genotype 4.3.1.1 used for Bayesian analysis. Download Table S4, XLSX file, 0.01 MB. Copyright © 2021 Sajib et al. 2021 Sajib et al. https://creativecommons.org/licenses/by/4.0/ This content is distributed under the terms of the Creative Commons Attribution 4.0 International license.

### Validation of MAMA.

**(i) DNA preparation.** To validate the assay, we selected an additional 38 AzmR (*Salmonella* Typhi, *n* = 32; *Salmonella* Paratyphi A, *n* = 06) and 62 randomly selected non-AzmR (*Salmonella* Typhi, *n* = 48; *Salmonella* Paratyphi A, *n* = 14) isolates isolated between 2016 and 2018 in Bangladesh. All the isolates (*n* = 113, including 13 from Hooda et al. [14]) selected for validation (see Table S1) were subjected to cell lysis by a simple boiling method. In brief, a single colony from MacConkey agar (Oxoid, Thermo Scientific) was suspended in a 1.5-ml microcentrifuge tube containing 200 μl of sterile DNase-free water. The tube was placed in a heating block at 100°C for 20 min and then centrifuged at 12,000 rpm for 5 min. The supernatant was used for downstream applications.

**(ii) Duplex PCR.** The total volume of the duplex PCR assay was 25* *μl, with 5× master mix (Hot FIREPol; Solis BioDyne, Tartu, Estonia). The final concentrations of the primers were 0.1 and 0.8 μM for the *parC* and *acrB* genes, respectively. Amplification was performed in a thermal cycler (ProFlex 3 × 32; Thermo Scientific) (Fig. 5A). The thermocycling conditions are shown in Fig. 5A. Amplified PCR products were run on 2% agarose gel (Invitrogen, Carlsbad, CA) at 100 V for 60 min and visualized on a Bio-Rad Gel Doc XR+ (Bio-Rad, Richmond, CA).

### Statistical analysis.

R 4.0.0 base functions and ggplot2, dplyr, epiR, and map packages were used for the sensitivity and specificity tests and statistical analyses. A Wilcoxon rank sum test was used to compare the mean MICs of wild-type and mutant isolates.
