# Supplementary figures and images for: Tracking the Emergence of Azithromycin Resistance in Multiple Genotypes of Typhoidal Salmonella
Source: mBio. 2021 Feb 16;12(1):e03481-20. doi: 10.1128/mBio.03481-20 (PMC8545119; doi:10.1128/mBio.03481-20)

FIG S1

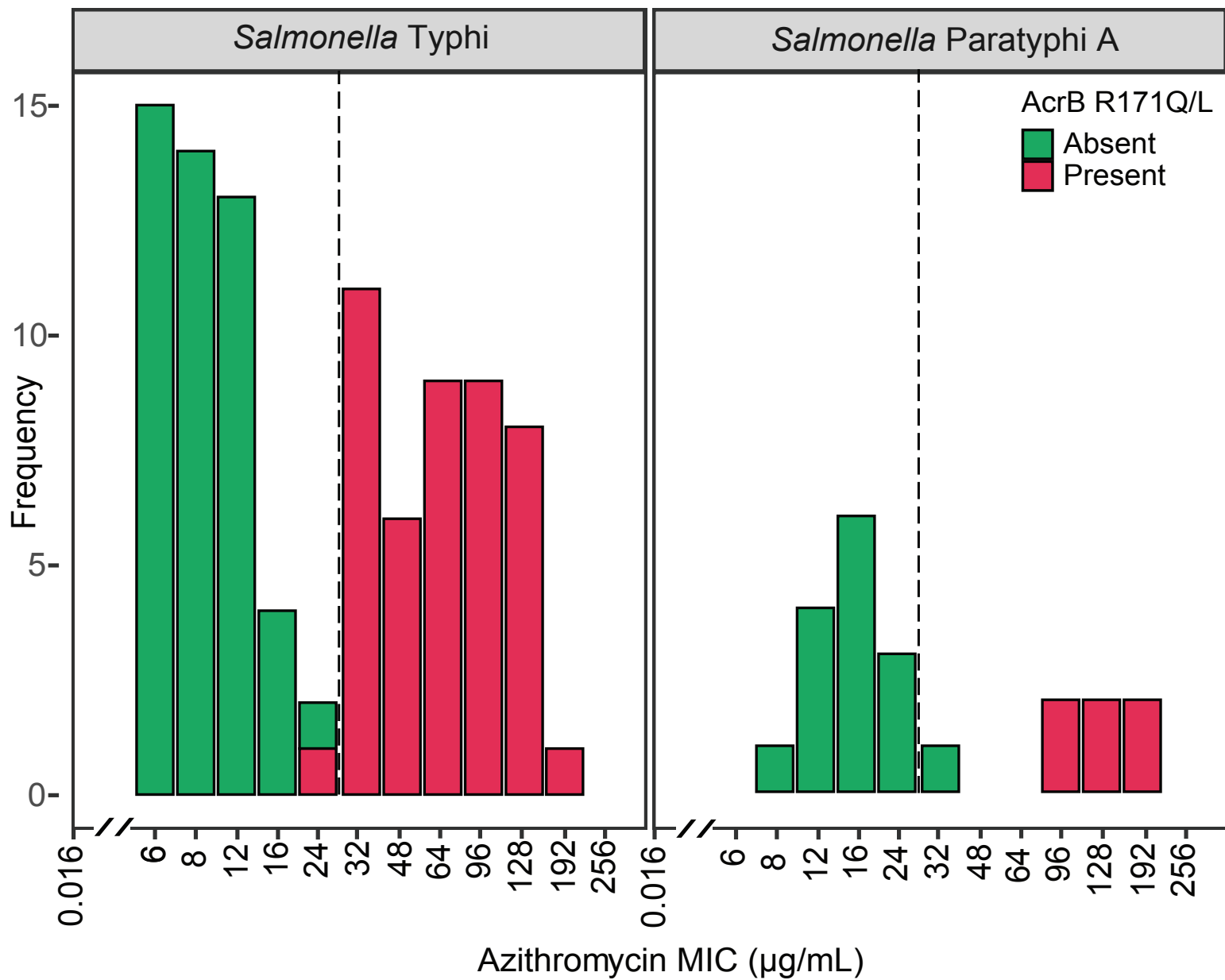

Supplement: FIG S1 [file mbio.03481-20-sf001.pdf]

FIG S2

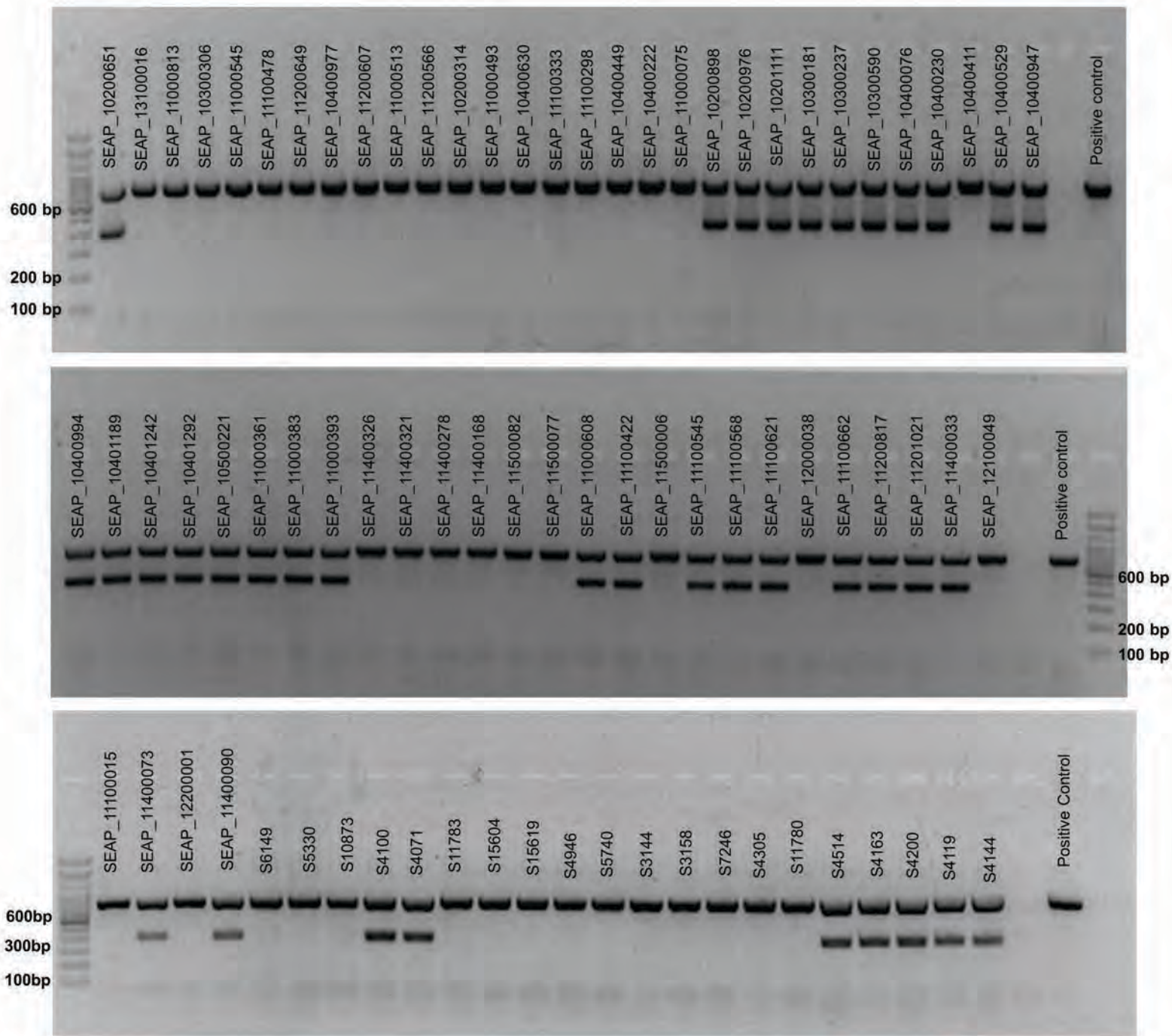

Supplement: FIG S2 [file mbio.03481-20-sf002.pdf]

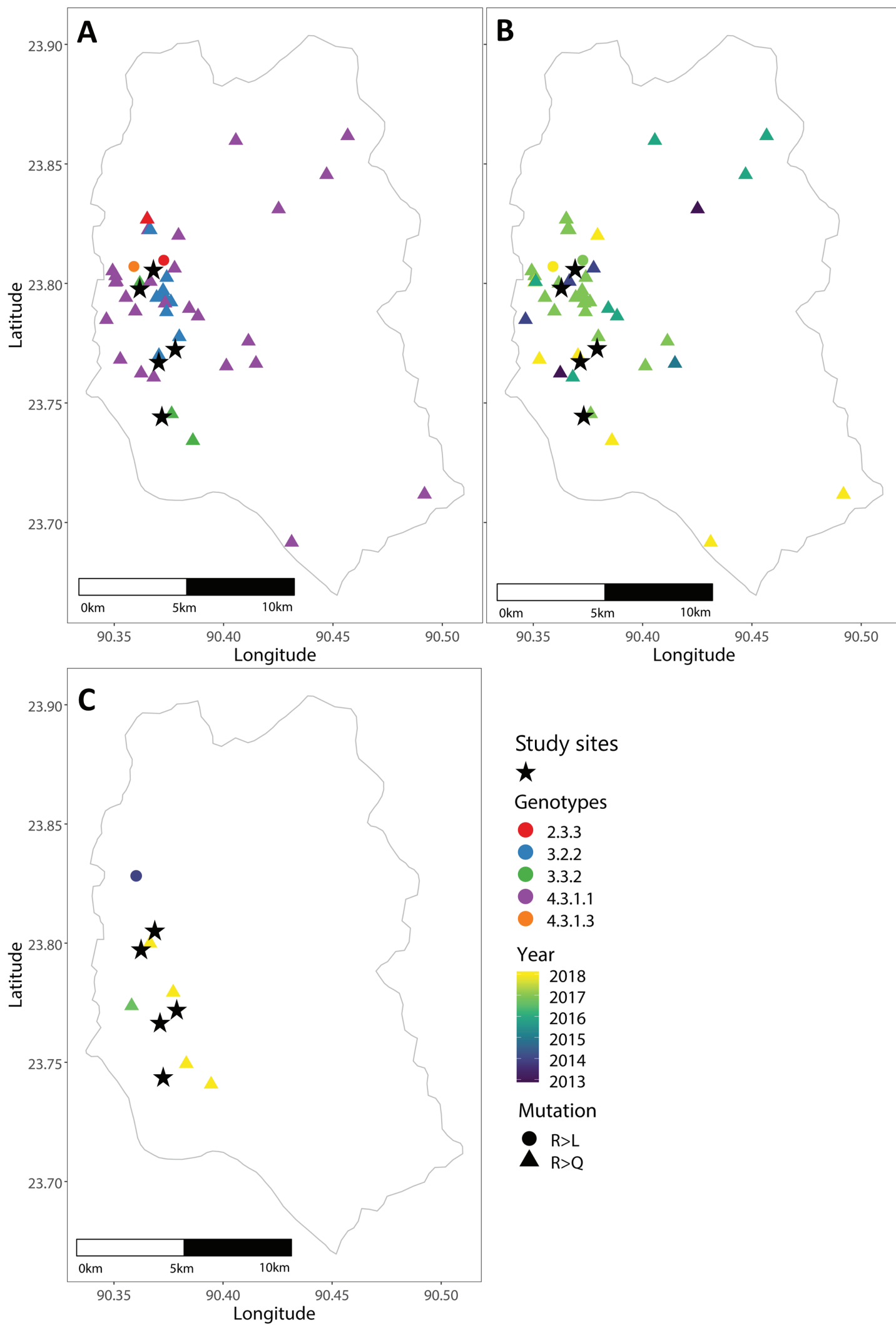

Supplement: FIG S3 [file mbio.03481-20-sf003.pdf]
